# Comprehensive Comparison of Chemical Composition and Antioxidant Activity of *Panax ginseng* Sprouts by Different Cultivation Systems in a Plant Factory

**DOI:** 10.3390/plants11141818

**Published:** 2022-07-10

**Authors:** Kye Man Cho, Hee Yul Lee, Du Yong Cho, Jea Gack Jung, Min Ju Kim, Jong Bin Jeong, Seong-Nam Jang, Ga Oun Lee, Han-Sol Sim, Min Ji Kang, Ye Lin Kim, Ji Hyun Lee, Sooyeon Lim, Ki-Ho Son

**Affiliations:** 1 Department of GreenBio Science, Agri-Food Bio Convergence Institute, Gyeongsang National University, Jinju-si 52725, Korea; kmcho@gnu.ac.kr (K.M.C.); wjdald99@nate.com (H.Y.L.); endyd6098@naver.com (D.Y.C.); jjbbkk5612@hanmail.net (J.G.J.); minju4492@naver.com (M.J.K.); love_no_ri@naver.com (J.B.J.); riyung@naver.com (G.O.L.); thfdl2828@naver.com (H.-S.S.); akflwl21@naver.com (M.J.K.); dpflskol@naver.com (Y.L.K.); 2 Division of Horticultural Science, Gyeongsang National University, Jinju-si 52725, Korea; tjdska1346@naver.com; 3 National Institute of Horticultural and Herbal Science, Rural Development Administration, Wanju-gun 55365, Korea; leejh80@korea.kr (J.H.L.); sylim84@korea.kr (S.L.)

**Keywords:** ginseng, ginsenoside, free amino acid, medicinal plant, vertical farm

## Abstract

In this study, the primary (such as amino acids, fatty acids, and minerals) and secondary (including ginsenosides, phenolic acids, and flavonols) metabolites and antioxidant effects of Panax ginseng sprouts (PGSs) by different cultivation systems, such as soil–substrate cultivation (SSC) and deep-water cultivation (DWC), in a plant factory has been observed. There was no significant difference in the total fatty acid (FA) contents. Particularly, the major FAs of PGSs were palmitic acid (207.4 mg/100 g) of saturated FAs and linoleic acid (397.6 mg/100 g) and α-linolenic acid (222.6 mg/100 g) of unsaturated FAs in the SSC system. The values of total amino acids were all higher in SSC than in DWC. In the case of ginsenosides, the total protopanaxtriol product was 30.88 mg/g in SSC, while the total protopanaxdiol product was 34.83 mg/g in DWC. In particular, the values of total phenolic acids and total flavonols were 133.36 and 388.19 ug/g, respectively, and SSC had a higher content than DWC. In conclusion, the SSC system was shown to be higher in nutritional constituents and antioxidant activities in soil cultivation, suggesting that PGS with SSC has a positive effect on the quality of PGS in a plant factory.

## 1. Introduction

Smart farming comprises an agricultural system that can cultivate crops in an optimal growth environment by using Information and Communication Technologies (ICTs) to offer great potential for improving efficiency, effectiveness, and productivity for crops [1]. Thus, smart farming is a new trend in agriculture to manage crops in controlled environments such as greenhouses and plant factories, which enables the control of optimal parameters. Moreover, severe outdoor environments that impact crop production and quality can be avoided with a complete management of growth conditions such as temperature, humidity, and lighting [2].

Ginseng (*Panax ginseng* C.A. Meyer) is used as an important medicinal plant in Asian countries such as Korea, China, and Vietnam. *Panax ginseng* sprouts (PGS) have been commercially cultivated as a medicinal vegetable in Korea due to their relatively short cultivation period and high amounts of bioactive compounds, including ginsenosides and amino acids [3]. Many studies recently reported that the content of ginsenosides of PGSs is higher in the leaves than in the root when cultivated for the same duration, and 1-year-old ginseng leaves have the highest ginsenoside content [4]. Production of ginseng sprout leaves has risen interest due to it being an excellent source for the food industry. Approximately 3 to 6 weeks of PGSs can be harvested, and they can make a high economic profit. Therefore, PGS can be produced in plant factories with artificial light (PFALs), which completely controls the environmental cultivation conditions and makes it possible to grow year-round in PFALs with a high quality of PGS [5,6].

Recently, PGS has been grown in hydroponic systems with short-term cultivation in a closed-type plant production system. These hydroponically grown PGS leaves from the plant factory can be recognized as an environmentally friendly natural food [7]. However, little research has been conducted on the nutritional constituents and antioxidant activities of hydroponically grown PGSs, and studies have not reported on PGS cultivated in the same conditions, such as light, temperature, place, etc., except for nutrient supply methods; therefore, it is necessary to explore the effect of different cultivation systems, such as soil–substrate cultivation (SSC) and deep-water cultivation (DWC), on PGSs to compare proper growth methods for cultivating PGSs in PFALs.

## 2. Results and Discussion

### 2.1. Comparison of Fatty Acid Content in Different Cultivation Systems

Table 1 compares the fatty acid (FA) contents dependent on two different cultivation systems. Among the saturated FAs, the content of palmitic acid was the highest (207.4 and 238.5 mg/100 g for SSC and DWC, respectively). The total saturated FA content was also higher for deep-water cultivation (329.3 mg/100 g) than for substrate cultivation (268.2 mg/100 g). In previous studies, Hwang et al. [6] and Kim et al. [5] studied PGSs cultivated in plant factories. However, Hwang et al. [6] and Kim et al. [5] did not report on FA contents either shoot or root. We analyzed the FA content of the entire whole PGS plant (shoot and root parts). Among the unsaturated FAs, linoleic acid exhibited the highest content (397.6 and 390.7 mg/100 g for soil and hydroponic cultivation, respectively). In addition, the α-linolenic acid content was notably high (222.6 and 176.0 mg/100 g for SSC and DWC, respectively). The total unsaturated Fas content was higher for SSC than for DWC. The overall total FA content was higher for SSC (950.6 mg/100 g) than for DWC (947.6 mg/100 g), although the difference was not significant.

Zhang et al. [8] previously reported that linoleic acid, among the 19 FAs in ginseng root, is the most predominant in the three *Panax* species, including *P. ginseng*, *P. notoginseng*, and *P. quinquefolus*, accounting for 43% to 53% of total FAs. Recently, in the case of PGSs produced by SSC systems in plant factories, it was reported that linoleic acid accounted for more than 50% of unsaturated FAs, which was similar to this result [5]. Linoleic acid is known to play a role in biological activities in plant tissue cultures and is a key enzyme of oxylipin biosynthesis [9]. In this study, the linoleic acid content among the unsaturated FAs was high for the SSC and DWC systems, based on the fact that a high level of biological activity is predicted for PGSs. 

### 2.2. Comparison of Free Amino Acid Contents in Different Cultivation Systems

Table 2 presents the PGS free amino acid (FAA) contents depending on the cultivation methods. The overall total FAA content was approximately two times higher for SSC (3592.36 mg/100 g) than for DWC (1942.23 mg/100 g). Among the non-essential amino acids (NEAAs), the proline content was 172.58 and 37.69 mg/100 g for the SSC and DWC systems, respectively. The contents of aspartic acid-NH_2_ and glutamic acid were approximately three times higher for SSC (241.67 and 165.22 mg/100 g, respectively) than for DWC (131.57 and 56.96 mg/100 g, respectively). The values of aspartic acid and γ-aminobutyric acid were also higher for SSC (241.67 and 247.75 mg/100 g, respectively) than for the DWC (102.71 and 163.38 mg/100 g, respectively). In particular, the highest NEAA content was exhibited by arginine (1365.53 and 746.80 mg/100 g for SSC and DWC, respectively). Essential amino acids must be consumed because they cannot be synthesized in the body. Among the essential amino acids (EAAs), valine and phenylalanine contents were notably high, while the total essential amino acid content was higher for SSC (599.53 mg/100 g) than for DWC (377.37 mg/100 g). Kim et al. [5] reported that the FAA amount in ginseng was higher in the plant parts above than below the ground. In this study, the DWC plants were grown with their roots in nutrient-enriched water. For this reason, it was expected that the FAA contents of DWC would be more likely to be eluted with water compared to SSC during the cultivation period, and therefore, the contents were less than the FAA contents of SSC. Moreover, it is necessary to perform more studies.

In the case of PGSs produced by the SSC system in a plant factory, Kim et al. [5] recently reported that aspartic acid, aspartic acid-NH_2_, glutamic acid, γ-aminobutyric acid, and arginine were major compounds of FAAs. Kim et al. [10] previously reported that arginine and histidine are the amino acids engaged in plant growth. For the SSC system, their contents were higher than for the DWC system, based on which it is anticipated that SSC of PGSs might contribute to the development of foods for growing children. In addition, γ-aminobutyric acid (GABA), among the FFAs, is the most abundant essential amino acid in raw and various processed ginseng products [11,12]. Furthermore, GABA is an inhibitory neurotransmitter in the central nervous system, and it can be considered as an important indicator in the quality control of different ginseng products [3,12].

### 2.3. Comparison of Mineral Contents in different Cultivation Systems

The supplementary data in Table 1 present the minerals of PGS contents dependent on the different cultivation methods, such as SSC and DWC. In the case of SSC, the phosphorus (P) and sulfur (S) contents were 5.12 and 2.66 mg/100 g, respectively, and although the values were slightly higher for the DWC, we observed no significant differences. However, calcium (Ca) and magnesium (Mg) showed higher contents in DWC (5.12 and 3.37 mg/100 g, respectively) than in soil cultivation (3.24 and 2.35 mg/100 g, respectively). Both for the SSC and DWC systems, the kalium (K) content was the highest among the minerals (31.45 and 27.53 mg/100 g, respectively). 

The main minerals of ginseng are known as P, Ca, K, and Mg [13]. Kim et al. [5] reported recently that P, S, Ca, K, and Mg of produced PGSs in plant factories were the major minerals. The comparison of the impact of dietary salt and potassium intake on blood pressure and obesity were reported as a relatively low risk of hypertension even upon a high intake of dietary Na in the case of high dietary K intake [14]. 

### 2.4. Comparison of Phytochemical Contents of Different Cultivation Systems

#### 2.4.1. Ginsenosides

**Figure 1:**
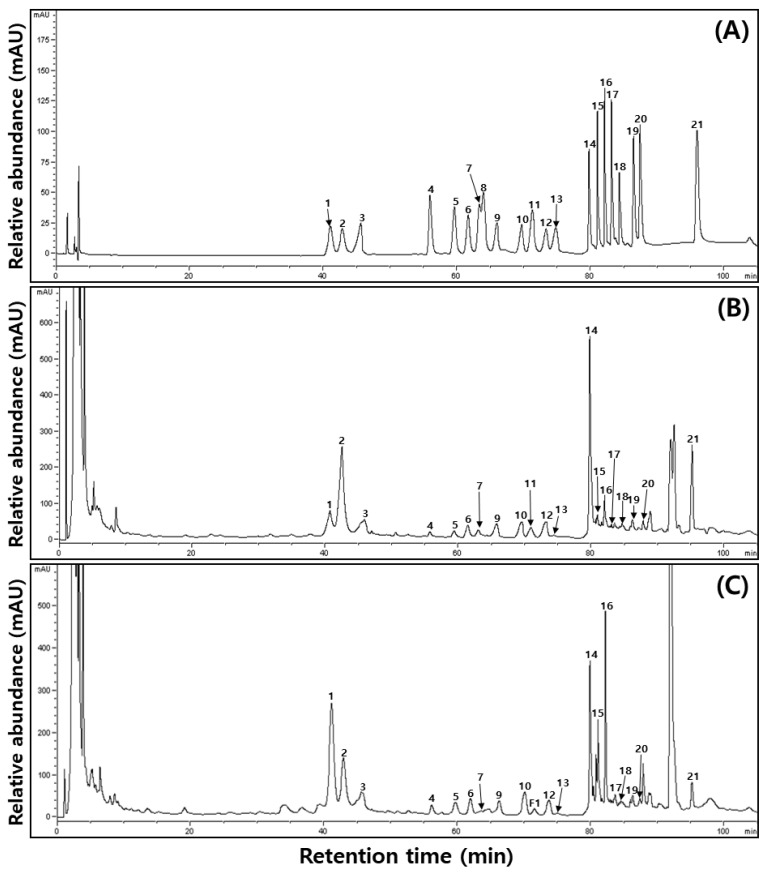
The typical HPLC chromatogram of PGS in different cultivation systems in a plant factory: (**A**) 21 ginsenoside standards; (**B**) soil–substrate cultivation; (**C**) deep-water cultivation. (1) Ginsenoside Rg1, (2) Re, (3) Ro, (4) Rf, (5) F5, (6) F3, (7) Rg2, (8) Rh1, (9) Rb1, (10) Rc, (11) F1, (12) Rb2, (13) Rb3, (14) Rd, (15) Rd2, (16) F2, (17) Rg3, (18) PPT, (19) Compound K, (20) Rh2, and (21) PPD.

Figure 1 and Table 3 present the ginsenoside contents in PGSs based on the different cultivation methods. The total ginsenoside content was 65.15 and 66.01 mg/g for the SSC and DWC systems, respectively, with the latter systems showing a higher content, although no statistically significant difference was observed. The major ginsenoside derivatives (more than 9 mg/g) were ginsenosides Re and Rd in the SSC systems and ginsenosides Rg1, Re, and F2 in the DWC systems. For the protopanaxtriol (PPT) compounds, the Rg1 content was 4.11 and 12.63 mg/g for SSC and DWC, respectively, showing approximately three times higher content for the DWC methods. In contrast, the Re content was two times higher for SSC (19.18 mg/g) than for DWC (9.42 mg/g). The ginsenosides Rf and F5 showed approximately two times higher content for hydroponic cultivation, while F3 showed no significant difference between the two cultivation methods. However, the values of Rg2 and F1 were approximately two to three times higher for SSC. Ginsenoside Rh1 could not be detected in any cultivation methods. The ginsenoside Rb1 among the protopanaxdiol (PPD) compounds was present at a higher content for SSC (3.32 mg/g) than for DWC (2.80 mg/g). The values of ginsenoside Rd2, F2, and PPT were approximately two times higher for DWC (6.42, 9.61, and 1.05 mg/g, respectively) than for SSC (2.34, 4.02, and 0.55 mg/g, respectively). However, the ginsenoside Rd, Rg3, and compound K (C.K) contents were approximately two times higher for SSC (12.65, 0.55, and 1.27 mg/g, respectively) than for DWC (6.87, 0.38, and 0.78 mg/g, respectively). 

The first attempt to isolate ginsenosides began in the year 1963 [15]. After that, there were approximately 200 ginsenosides were found from the *Panax* species [16]. Ginsenosides are typically classified into two groups based on chemical structure as follows: four-cyclic structure dammarne type and five-cyclic structure oleanane type. In addition, ginsenosides of the dammarne type are divided into two groups: PPD and PPT [16]. Farnesyl diphosphate is synthesized as squalene in the isoprenoid pathway [17], and as phyrosterol and triterpene through the squalene epoxidase reaction and catalysis of oxidosqualene cyclases. β-amyrin is synthesized, which is converted into ginsenosides through hydroxylation and glycosylation [18]. The cytochrome P450S enzyme was reported to be involved in the hydroxylation of carbon 6 of the PPD type and is converted to PPD [19], and glycosyltransferase enzymes were reported to act on the backbones of PPD and PPT to cause glycosylation [20,21,22]. It is presumed that various ginsenoside derivatives are synthesized through this saccharification process. Kim et al. [23] and Hwang et al. [6] recently reported that the ginsenosides Re and Rd of produced PGSs in plant factories were the major ginsenoside compounds. The ginsenoside Rg1, Re, Rd, and F2 have various biological effects, including antioxidative, antidiabetes, anti-inflammatory, antidementia, and antitumor activities [24,25,26,27,28]. Compared with the earlier reported data associated with the plant factory, our results were higher than in below-ground PGSs (25.81–34.19 and 37.39 mg/g) [5,6]. In contrast, they were lower than the above-ground PGSs (61.31–110 and 101.73 mg/g) [5,6]. In addition, there was a large amount contained in soil-cultured PGSs [29] and mountain-cultivated ginseng sprouts [24]. Our results are similar to those of prior studies, which showed that Rg1 and Re were the major ginsenoside compounds in hydroponic-cultured ginseng and soil-cultured ginseng, respectively [5,6,30]. We expected identified Rg1 and Re to be the critical compounds between the DWC and the SSC systems in a plant factory.

#### 2.4.2. Phenolic Acids and Flavonols

Table 4 compares the phenolic acid and flavonol contents in PGSs dependent on the cultivation method. The SSC system generally showed higher contents across different phenolic acids including protocatechuic acid (11.22 μg/g), chlorogenic acid (32.31 μg/g), *p*-hydroxybenzoic acid (11.83 μg/g), and benzoic acid (62.16 μg/g), although DWC showed a slightly higher gallic acid content (10.20 μg/g). The content of *p*-coumaric acid was not detected for SSC, while DWC showed 1.23 μg/g for the compounds. Conversely, the value of the veratric acid compound was not detected for DWC, while its content was 2.53 μg/g for SSC. Among the flavonols, the content of catechin was approximately four-fold higher for SSC (81.22 μg/g) than for DWC (21.30 μg/g), and the content of quercetin was also higher for SSC (177.79 μg/g) than for DWC (112.94 μg/g). In addition, the contents of epicatechin, naringin, and naringenin were higher for SSC than for DWC, while the content of epigallocatechin was higher for DWC (115.19 μg/g) than for SSC (90.52 μg/g). The following compounds were not detected as follows: vanillin, rutin, and catechin gallate.

Previously, ten phenolic/or flavonoid profiles (such as salicylic acid, vanillic acid, ascorbic acid, *p*-coumaric acid, ferulic acid, caffecic acid, gentisic acid, *p*-hydorxybenzoic acid, maltol, cinnamic acid, protocatechuic acid, syringic acid, and quercetin) were investigated in fresh and/or processed ginseng/or ginseng seed [16,24,28,31]. Chung et al. [32] reported that Korean ginseng contains *p*-coumaric acid, which is especially highest in the leaves. In addition, this compound was reported to be high in the leaves of hydroponically cultivated PGSs [33]. In this study, the catechin and quercetin of ginseng were lower than green tea and white and red onions [34,35]. According to other reports, quercetin in ginseng roots was either not detected or detected in minute quantities [32]. Our results showed that quercetin was the main flavonol in PGSs cultured in a plant factory. These PGSs are commercially significant compared to ginseng root. Recently, Kim et al. [5] reported that among the phenolic acids and flavonols detected in the above-ground part of sprouted ginseng, chlorogenic acid and quercetin were detected as the major compounds, respectively. Chlorogenic acid and quercetin also have multiple biological effects, including antioxidative, antidiabetes, anti-inflammatory, antidementia, and antitumor activities [5,24,36,37]. The synthesis of phenolic acids was carried out through the shikimate pathway. In this process, amino acids (such as phenylalanine, tyrosine, and proline) are required for synthesis [38]. First, it is presumed that the synthesis of phenolic acids is relatively low because DWC has a lower content than SSC. Second, it was assumed that there was a possibility that the soluble phenolic acids would be eluted into water when grown for a long time. Further research on this is necessary.

### 2.5. Comparison of Total Phenolic Contents and Total Flavonoid Contents in Different Cultivation Systems

**Figure 2:**
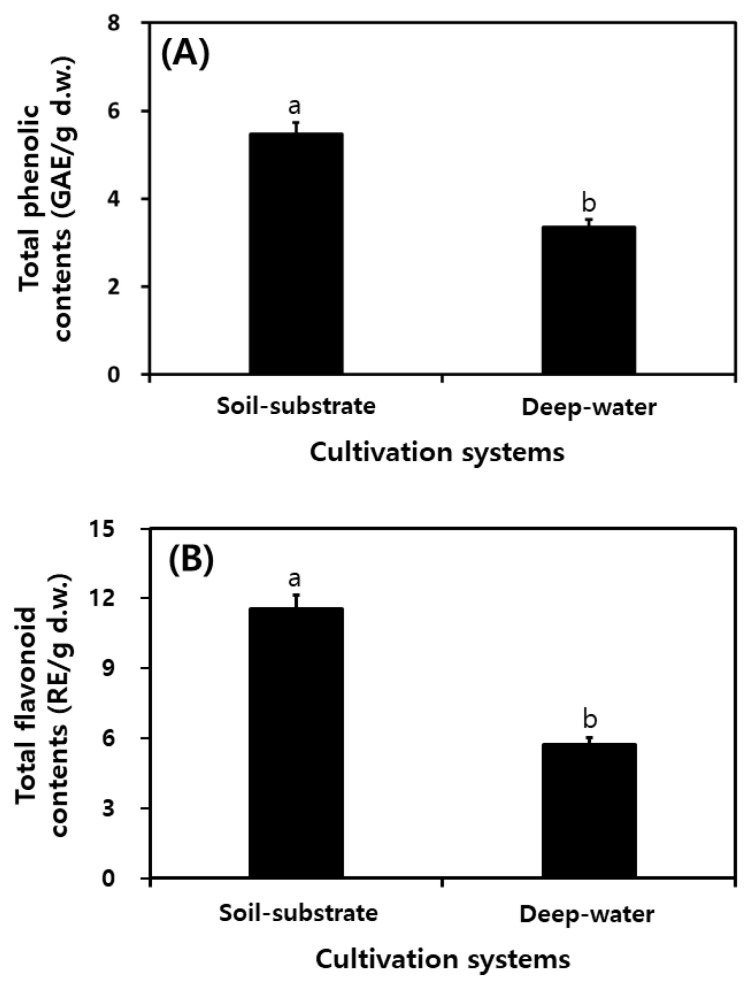
Comparison of total phenolic and total flavonoid contents of PGS in different cultivation systems in a plant factory. (**A**) Total phenolic contents; (**B**) total flavonoid contents. All values are presented as the mean ± SD of pentaplicate determination. Different letters correspond to the significant differences related to the processing steps using Tukey’s multiple test (*p* < 0.05).

Figure 2 shows the values of total phenolic content (TPC) and total flavonoid content (TFC) in PGSs based on the different cultivation systems. TPCs were 5.46 and 3.35 mg/g for SSC and DWC, respectively. TFC was approximately two-fold higher for SSC at 11.56 mg/g than for DWC at 5.74 mg/g. 

TPCs and TFCs of DWC and SCC were high compared with previous studies regarding hydroponic-cultured ginseng [30,39] and soil-cultured ginseng [32,40]. Phytochemicals, including phenolic acids, flavonoids, isoflavones, and flavonols, are widely present in various plants and are known as representative antioxidants derived from natural products [21,25]. Many studies have previously reported that potential antioxidant activities are positively correlated with TPC and TFC [5,24,32,33,41,42]. We considered that these compounds (including TPC and TFC) affect radical scavenging activities and reduce the power of the antioxidants. In this study, TPCs and TFCs were higher in SSC than in DWC. Therefore, it was presumed that the SSC system had higher antioxidant activities than the DWC system. We considered that these compounds (including TPCs and TFCs) affected radical scavenging activities and reduced the power of the antioxidants. In this study, TPCs and TFCs were higher in SSC than in DWC. Therefore, it was presumed that the SSC system had higher antioxidant activities than the DWC system. It was also presumed that the SSC system had higher antioxidant activities than the DWC system. Generally, berries among fruits and vegetables present rich antioxidant phenolics [43]. 

### 2.6. Comparison of Antioxidant Effects in different Cultivation Systems

**Figure 3:**
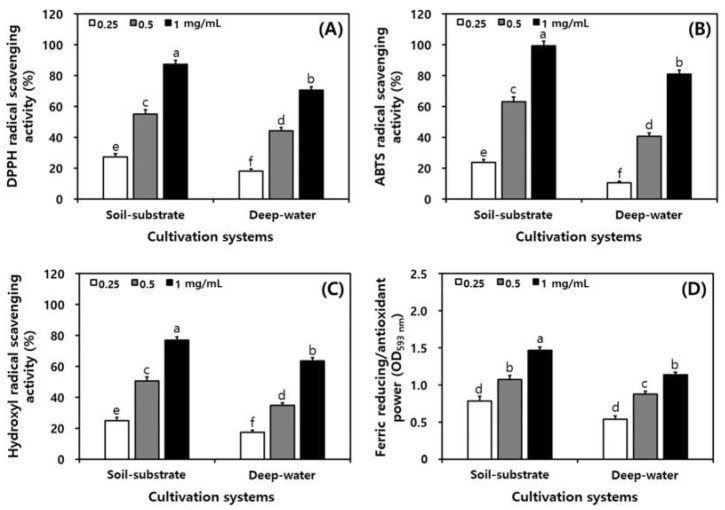
Comparison of antioxidant activity of PGS in different cultivation systems in a plant factory. (**A**) DPPH radical scavenging activity; (**B**) DPPH radical scavenging activity; (**C**) hydroxyl radical scavenging activity; (**D**) ferric reducing/antioxidant power. All values are presented as the mean ± SD of pentaplicate determination. Different letters correspond to the significant differences related to the processing steps using Tukey’s multiple test (*p* < 0.05).

The 2,2-diphenyl-1-picrylhydrazyl (DPPH), 2-2’-azino-bis (3-ethylbenzothiazoline-6-sulfonic acid) diammonium salt (ABTS), and hydroxyl radical scavenging activities and ferric reducing/antioxidant power (FRAP) assay of PGSs are presented in Figure 3. The DPPH radical scavenging activity of 1 mg/mL of PGSs was higher for SSC (87.62%) than for DWC (70.56%) (Figure 3A). The ABTS radical scavenging activity was also higher for SSC (63.07% in 0.5 mg/mL and 99.34% in 1 mg/mL of PGSs) than for DWC (40.68% in 0.5 mg/mL and 81.04% in 1 mg/mL of PGSs) (Figure 3B). The hydroxyl radical scavenging activity was 25.01% and 17.29% in 0.25 mg/mL of PGSs for SSC and DWC, respectively (Figure 3C). The FRAP reducing power was 1.468_OD593 nm_ for SSC and was 1.138_OD593 nm_ for DWC in 1 mg/mL of PGSs (Figure 3D).

We are confident that the antioxidant properties of the 50% ethanol extracts of PGSs produced in the SSC and DWC systems correlate with the profiles and contents of ginsenoside, phenolic acid, and flavonol compounds [44,45]. However, our findings evaluated for the first time the antioxidant properties of PGSs produced from SSC and DWC systems in a plant factory. Overall, the produced PGS samples by the SSC techniques may be considered an excellent natural antioxidant source for nutraceutical agents. Thus, the high antioxidant activity of PGSs might be attributed to the individual antioxidant activities of ginsenosides, phenolic acids and flavonols, especially ginsenoside Re and Rd, chlorogenic acid, and quercetin.

## 3. Materials and Methods

### 3.1. Chemicals and Instruments

The Folin–Ciocalteu reagent for measuring TPC and diethylene glycol for measuring TFC were purchased from Sigma-Aldrich Chemical (St. Louis, MO, USA). The reference standards for phenolic acids (such as gallic, protocatechuic, chlorogenic, *p*-hydroxybenzoic, vanillic, *p*-coumaric ferulic, benzoic, veratric, and *t*-cinnamic acids) and flavonols (including epigallocatechin, catechin, epicatechin, epigallocatechin gallate, vanillin, rutin, catechin gallate, quercetin, naringin, naringenin, and formononetin) were purchased from Sigma-Aldrich, while those for saponin (ginsenoside Rb1, Rb2, Rb3, Rg1, Rg2, Rg3, Rd, Rd2, F1, F2, F3, F5, Rh1, Rh2, Rc, Re, Rf, Ro, C.K, PPD, and PPT) were purchased from KOC Biotech Co., Ltd. (Daejeon, Korea). Other materials including DPPH, ABTS, thiobarbituric acid (TBA), and trichloroacetic acid (TCA) were also purchased from Sigma-Aldrich Chemical. All other reagents purchased were of the highest grade. HPLC-grade H_2_O, acetonitrile, and methanol were purchased from Fisher Scientific (Fairlawn, NJ, USA). 

The FA analysis was performed using a gas chromatography (GC) 7980 system (Agilent Technologies Inc., Wilmington, DE, USA) containing a flame ion detector (FID) and SP-2560 capillary column (100 m × 0.25 mm, 0.25 μm film thickness; Sigma-Aldrich Co., St. Louis, MO, USA). The contents of FAAs and minerals were measured using automatic amino acid analyzer (Hitachi High-Technologies Corp., Tokyo, Japan) and an inductively coupled plasma spectrometer (ICP, NexION 350 ICP MS, PerkinElmer Inc., Waltham, MA, USA), respectively. The analysis of phytochemicals (including ginsenosides, phenolic acids, and flavonols) was carried out using high-performance liquid chromatography (HPLC Agilent 1260 series Co., Forest Hill, Vic, Australia) with a diode array detector (DAD), a quaternary pump, autosampler, and: Tosoh Corp., Tokyo, Japan) and X-Bridge C18-RP column (for phenolic acids and flavonols: Waters Corp., Milford, MA, USA). The TPCs and TFCs and antioxidant assay were performed using UV–Vis absorption spectra on a Shimadzu Scientific Korea Corp. (UV-1800 240V, Seoul, Korea).

### 3.2. Growth of PGSs by Different Cultivation Methods

Based on the cultivation methods, smart farm cultivated PGSs were divided into two groups, such as SSC and DWC, and then grown for 4 weeks to be used in subsequent analyses. Seedlings were planted in trays (51 × 35 × 5 cm, (L × W × H)) containing a ginseng-exclusive medium (Shinsung Mineral Co., Ltd., Seongnam, Korea). These planted seedlings were cultivated in a Smart Farm Cube (Dream Cop., Sacheon, Korea) under natural red and blue LED light, temperature 20 ± 2 °C, and relative humidity 60 ± 5%, and CO_2_ 400 µmol mol^−1^. The PGSs using SCC and DWC were cultivated only one time. This is because the environment (light, temperature, and humidity etc.) can be maintained constantly in a plant factory.

### 3.3. Nutrient Compounds Analysis

#### 3.3.1. FA Analysis

For FA analysis, the method by Hwang et al. [41] was used with slight modifications. Briefly, 3 mL of 0.5 N methanolic NaOH was added to a test tube containing accurately weighed 1 g of sample. The sample in the test tube was heated at 100 °C for 10 min for fatty acid and glycerol hydrolysis. Next, 2 mL of BF_3_ was added into the tubes, stirred, and then the mixture was heated for a further 30 min for fatty acid esterification. At the completion of methyl esterification, 1 mL of isooctane was added into the tubes, and the mixture was left to stand for a while, after which only the isooctane layer was collected for dehydration with anhydrous sodium sulfate and filtered through a 0.45 μm membrane filter. Next, GC analysis was performed with N_2_ gas as a mobile phase and at a flow rate of 1 mL/min. The temperature of the oven was maintained at 140 °C for 5 min and then raised by 20 °C per min until reaching 180 °C. This temperature was maintained for 2 min and then raised by 50 to 230 °C and maintained for 35 min. The temperatures of the injector and the FID detector were set to 220 and 240 °C, respectively.

#### 3.3.2. FAAs Analysis

For FAAs analysis, 1 g of sample was mixed with 4 mL of distilled water and stirred. After 1 h of hydrolysis at 60 °C, 1 mL of 10% 5-sulfosalicylic acid was added, and the mixture was left to stand for 2 h at 4 °C for protein precipitation. Then, the samples were centrifuged for 3 min, and the supernatant was filtered through a 0.45 μm membrane filter, followed by vacuum concentration at 60 °C. The concentrated sample was dissolved in 2 mL of lithium citrate buffer (pH 2.2), and the solution was filtered through a 0.45 μm membrane filter. The filtrate was analyzed using an automatic amino acid analyzer [41].

#### 3.3.3. Mineral Analysis

For mineral analysis, 0.5 g of sample was weighed in a test tube, and 10 mL of 70% nitric acid solution was added to the tubes for subsequent high-pressure microwave digestion. Thereafter, the total volume was adjusted to 50 mL by adding distilled water for subsequent ICP analysis [24].

### 3.4. Phytochemical Compounds Analysis

#### 3.4.1. Extract Preparation

To analyze the phytochemicals, such as ginsenosides, phenolic acids, and flavonols, one gram of two powder samples was added to the 50 mL of 50% methanol, and the mixtures were stationarily extracted on a water bath at 40 ± 2 °C for 1 h. The extracts were filtered through a 0.45 µm membrane filter to recover the supernatant; this process was repeated two times. Afterward, each extract–supernatant was concentrated and dried using a rotary evaporate. Finally, the dried extracts were added to the 2 mL of acetonitrile: H_2_O (8:2, *v*/*v*) and dissolved. The dissolved samples were filtered through a 0.45 µm membrane filter for analysis of HPLC [24].

#### 3.4.2. Ginsenoside Compounds Analysis

The ginsenosides were analyzed using HPLC, with a slightly modified version of the method of Lee et al. [24]. The flow rate was maintained at 1.0 mL/min, and 10 μL of the sample was injected for analysis. For the solvent in the mobile phase, solvent A (HPLC water) and B (acetonitrile) were used. The analysis was carried out at 203 nm based on solvent B (10 min at 19%, 15 min at 20%, 40 min at 23%, 42 min at 30%, 75 min at 35%, 80 min at 70%, 90 min at 90%, and 100 min at 90%). 

#### 3.4.3. Phenolic Acid and Flavonol Compounds Analysis

The contents of phenolic acid and flavonol derivatives were measured using the HPLC method of Lee et al. [24] with slight modification. To determine the phenolic acids and flavonols, the flow rate, injection volume, and column oven temperature were adjusted at 1 mL/min, 20 µL, UV 270 nm (for flavonols) and UV 280 nm (for phenolic acids), and 30 °C, respectively. The mobile phase consisted of H_2_O with 0.5% acetic acid (solution A) and MeOH (solution B) with an elution program as follows: 10 min at 15% B, 15 min at 5% B, 20 min at 15% B, 25 min at 5% B, 30 min at 10% B, 35 min at 50% B, 40 min at 50% B, 45 min at 60% B, 55 min at 80% B, and 60 min at 90% B.

### 3.5. TPCs and TFCs Analysis

#### 3.5.1. Preparation of Samples for TPCs and TFCs

The extracts were prepared as follows: The dried powder samples (1 g) with 50% ethanol 20 mL was extracted at room temperature for 12 h using the multi vortex-genie (SI-M256, Multi Vortex-Genie, Scientific Industries, USA). The extract was filtered through a 0.45 µm membrane filter to recover the supernatant; this process was repeated two times. To prepare the extract–powders, each extract–supernatant was concentrated using a rotary evaporator and then freeze-dried. Finally, the extract–powders were dissolved to 0.25, 0.5, and 1.0 mg/mL by 50% EtOH and the filtered. The extract–supernatants and extract–powders were tested for analyzed TPCs and TFCs and antioxidant effects, respectively [24].

#### 3.5.2. TPCs Analysis

To measure the TPCs, a slightly modified version of the Folin Denis method [9] was used. Briefly, 0.5 mL of the extract was placed in a test tube and supplemented with 0.5 mL of 25% Na_2_CO_3_ solution. The mixture was left to stand for 3 min and supplemented with 0.25 mL of 2 N Folin–Ciocalteu reagent. The mixture was left to stand for 1 h at 30 °C for coloration, and the absorbance of the resulting sample was measured using a spectrophotometer (Spectronic 2D). To estimate the total phenolic content, gallic acid was used to draw a standard calibration curve. The content was expressed as the means of triplicate experiments. The results are expressed in milligrams of gallic acid equivalent per gram on a dry weight basis (mg GAE·g^−1^ DW).

#### 3.5.3. TFCs Analysis

To measure the total flavonoid content, the method of Lee et al. [24] was used. Briefly, 0.5 mL of the extract was placed in a test tube and supplemented with 1.0 mL of diethylene glycol, followed by the addition of 0.01 mL of 1 N NaOH. The mixture was left to stand for 1 h in a constant-temperature water bath at 37 °C. The absorbance was measured at 420 nm using a spectrophotometer. To calculate the total flavonoid content, rutin was used to obtain a standard calibration curve. The content was expressed as the mean of triplicate experiments.

### 3.6. Antioxidant Activities

The antioxidant effects against DPPH, ABTS and hydroxyl radical scavenging activities and ferric reducing/antioxidant power (FRAP) were measured using the methods of those previously reported by Lee et al. [10], with slight modification. To evaluate the DPPH and ABTS assays, the diluted solution of the 50% EtOH extract–concentrates (0.2 mL or 0.1 mL) was mixed with the 1.5 × 10^−4^ Mm DPPH solution (0.8 mL) or the mixtures of 7 mM ABTS and 2.45 mM K_2_S_2_O_8_ solutions (0.9 mL), respectively. The final reaction products were determined at 525 or 730 nm using a UV–Vis spectrophotometer. The FRAP solution was prepared by mixing 300 mM acetate buffers (pH 3.6), 10 mM TPTZ in 40 mM HCl, and 20 mM FeCl_3_ in a ratio of 10:1:1. Prepared solution was preliminarily reacted at 37 °C for 15 min and after 0.05 mL diluted samples and 0.95 mL FRAP solution was mixed and reacted at 37 °C for 15 min. The reaction sample was measured at 593 nm.

### 3.7. Statistical Analysis

The nutrient compounds and antioxidant activities were expressed as the mean ± SD (standard derivation) of five measurements. The significant differences among samples were determined by Tukey’s multiple test (*p* < 0.05) using the Statistical Analysis System (SAS) software (ver. 9.4; SAS institute, Cary, NC, USA).

## 4. Conclusions

Ginseng has long been widely used as a medicinal crop. According to a recent study, the leaves of ginseng contain a higher amount of ginsenoside compounds that are not found in the roots. In this study, for the first time, cultivated PSGs using two systems, SSC and DWC, were compared in a plant factory. The value of total fatty acids was almost the same, but saturated and unsaturated fatty acid contents were higher in DWC and SSC, respectively. However, the contents of total amino acids, non-essential amino acids, and essential amino acids were all higher in SSC than in DWC. In addition, there was no significant difference in the mineral contents. The total ginsenoside content was similar. The PPT form had higher SSC and the PPD form had higher DWC, but there was no significant difference. In particular, the main ginsenosides were ginsenoside Re (19.18 mg/g) and Rd (12.65 mg/g) in SSC, but ginsenoside Rg1 (12.63 mg/g) was present in DWC. The total phenolic acids and flavonols were both higher in SSC than in DWC. Both compounds, chlorogenic acid (32.31 μg/g) of phenolic acids and quercetin (177.79 μg/g) of flavonols, were considerably higher in SSC than in DWC. The values of TPC and TFC were higher in SSC than DWC. Correspondingly, the DPPH, ABTS and hydroxyl radical scavenging activities and FRAP assay was higher in SSC than in DWC. From this result, it can be suggested that the SSC system is more effective than the DWC system in terms of nutrients and antioxidant activities of PGSs in plant factories. However, if additional studies about the DWC system are studied worked based on this result, it is considered that the PSGs cultured in the DWC system will improve the value as much as the SSC system. This result proposes that PGSs may contributed to enhancing the ginseng value, which can then be used as nutritional and functional ginseng products, such as foods, cosmetics, drugs, etc.

## Figures and Tables

**Table 1:** Comparison of fatty acid contents of PGS in different cultivation systems in a plant factory.

Contents ^1^ (mg/100 g d.w.)	Cultivation Systems	
	Soil–Substrate	Deep-Water
Saturated fatty acids		
Myristic acid (C14:0)	5.2 ± 0.26 a	4.9 ± 0.25 a
Palmitic acid (C16:0)	207.4 ± 10.37 ab	238.5 ± 11.93 a
Stearic acid (C18:0)	33.1 ± 1.66 b	56.6 ± 2.83 a
Arachidic acid (C20:0)	4.7 ± 0.24 b	6.8 ± 0.34 a
Behenic acid (C22:0)	9.5 ± 0.48 b	13.5 ± 0.68 a
Lignoceric acid (C24:0)	8.3 ± 0.42 a	9.0 ± 0.45 a
Total	268.2	329.3
Unsaturated fatty acids		
Palmitoleic acid (C16:1)	7.7 ± 0.39	Nd ^2^
Oleic acid (C18:1n9c)	35.6 ± 1.78 a	34.2 ± 1.71 a
Linoleic acid (C18:2c)	397.6 ± 19.88 a	390.7 ± 19.54 a
α-Linolenic acid (C18:3n3)	222.6 ± 1.13 a	176.0 ± 8.80 b
Eicosenic acid (C20:1)	3.6 ± 0.18 a	3.3 ± 0.17 a
Eicosadienoic acid (C20:2)	3.7 ± 0.19 a	3.6 ± 0.18 a
Eicosatrienoic acid (C20:3n3)	6.1 ± 0.31 a	5.7 ± 0.29 a
Tricosanoic acid (C23:0)	5.5 ± 0.28 a	4.8 ± 0.24 ab
Total	682.4	618.3
Total fatty acids	950.6	947.6

^1^ All values are presented as the mean ± SD of pentaplicate determination. Different letters correspond to the significant differences related to the processing steps using Tukey’s multiple test (*p* < 0.05). ^2^ nd: not detected.

**Table 2:** Comparison of free amino acid contents of PGS in different cultivation systems in a plant factory.

Contents ^1^ (mg/100 g d.w.)	Cultivation Systems	
	Soil–Substrate	Deep-Water
Non-essential amino acids (NEAAs)		
Proline	172.58 ± 8.63 a	37.69 ± 1.88 b
Aspartic acid	241.67 ± 12.08 a	102.71 ± 5.14 b
Serine	97.06 ± 4.85 a	66.51 ± 3.33 b
Aspartic acid-NH_2_	313.37 ± 15.67 a	131.57 ± 6.58 b
Glutamic acid	165.22 ± 8.26 a	56.96 ± 2.85 a
Sarcosine	5.49 ± 0.27 a	nd ^2^
Aminoadipic acid	9.53 ± 0.48 a	4.40 ± 0.22 a
Glycine	18.32 ± 0.92 a	12.50 ± 0.63 ab
Alanine	108.21 ± 5.41 a	86.98 ± 4.35 ab
Citrulline	24.81 ± 1.24 a	3.82 ± 0.19 b
α-aminobutyric acid	13.58 ± 0.68 ab	17.66 ± 0.88 a
Cystine	9.58 ± 0.48 a	8.45 ± 0.42 a
Tyrosine	71.33 ± 3.57 a	41.01 ± 2.05 b
β-alanine	28.28 ± 1.41 a	27.40 ± 1.37 a
β-aminoisobutyric acid	27.09 ± 1.35 a	16.25 ± 0.81 b
γ-aminobutyric acid	247.75 ± 12.39 a	163.38 ± 8.17 b
Aminoethanol	24.47 ± 1.22 a	20.90 ± 1.05 a
Hydroxylysine	2.80 ± 0.14 a	1.88 ± 0.09 b
Ornithine	45.41 ± 2.27 a	17.99 ± 0.90 b
3-methylhistidine	0.75 ± 0.04	nd ^2^
Arginine	1365.53 ± 68.28 a	746.80 ± 78.24 b
Total	2992.83	1564.86
Essential amino acids (EAAs)		
Threonine	72.59 ± 3.63 a	47.31 ± 2.37 b
Valine	102.81 ± 5.14 a	66.75 ± 3.34 b
Methionine	18.71 ± 0.94 a	16.28 ± 0.81 a
Isoleucine	68.73 ± 3.44 a	44.12 ± 2.21 b
Leucine	98.88 ± 4.94 a	54.89 ± 2.74 b
Phenylalanine	101.89 ± 5.09 a	52.47 ± 2.62 b
Lysine	90.05 ± 4.50 a	66.03 ± 3.30 b
Histidine	45.87 ± 2.29 a	29.52 ± 1.48 b
Total	599.53	377.37
Total amino acids	3592.36	1942.23

^1^ All values are presented as the mean ± SD of pentaplicate determination. Different letters correspond to the significant differences related to the processing steps using Tukey’s multiple test (*p* < 0.05). ^2^ nd: not detected.

**Table 3:** Comparison of ginsenoside contents of PGS in different cultivation systems in a plant factory.

Contents ^1^ (mg/g d.w.)	Cultivation Systems	
	Soil–Substrate	Deep-Water
Protopanaxtriol types		
Ginsenoside Rg1 (1)	4.11 ± 0.21 b	12.63 ± 0.63 a
Ginsenoside Re (2)	19.18 ± 0.96 a	9.42 ± 0.47 b
Ginsenoside Rf (4)	0.36 ± 0.02 b	0.61 ± 0.03 a
Ginsenoside F5 (5)	0.62 ± 0.03 b	1.26 ± 0.06 a
Ginsenoside F3 (6)	3.02 ± 0.15 a	2.98 ± 0.15 a
Ginsenoside Rg2 (7)	1.4 ± 0.07 a	0.45 ± 0.02 b
Ginsenoside Rh1 (8)	nd ^2^	nd
Ginsenoside F1 (11)	1.13 ± 0.06 a	0.62 ± 0.03 b
Protopanaxtriol (18)	1.06 ± 0.05 a	0.39 ± 0.02 b
Total	30.88	28.36
Protopanaxdiol types		
Ginsenoside Rb1 (9)	3.32 ± 0.17 a	2.80 ± 0.14 ab
Ginsenoside Rc (10)	2.80 ± 0.14 ab	3.13 ± 0.16 a
Ginsenoside Rb2 (12)	3.76 ± 0.19 a	2.69 ± 0.13 b
Ginsenoside Rb3 (13)	0.32 ± 0.02 b	0.41 ± 0.02 a
Ginsenoside Rd (14)	12.65 ± 0.63 a	6.87 ± 0.34 b
Ginsenoside Rd2 (15)	2.34 ± 0.12 b	6.42 ± 0.32 a
Ginsenoside F2 (16)	4.02 ± 0.20 b	9.61 ± 0.48 a
Ginsenoside Rg3 (17)	0.55 ± 0.03 a	0.38 ± 0.02 b
Compound K (19)	1.27 ± 0.06 a	0.78 ± 0.04 b
Ginsenoside Rh2 (20)	0.76 ± 0.04 a	0.69 ± 0.03 a
Protopanaxdiol (21)	0.55 ± 0.03 b	1.05 ± 0.05 a
Total	32.34	34.83
Oleanane types		
Ginsenoside Ro (3)	1.93 ± 0.10b	2.83 ± 0.14a
Total	1.93	2.83
Total ginsenosides	65.15	66.01

^1^ All values are presented as the mean ± SD of pentaplicate determination. Different letters correspond to the significant differences related to the processing steps using Tukey’s multiple test (*p* < 0.05). ^2^ nd: not detected.

**Table 4:** Comparison of phenolic acid and flavonol contents of PGS in different cultivation systems in a plant factory.

Contents ^1^ (μg/g d.w.)	Cultivation Systems	
	Soil–Substrate	Deep-Water
Phenolic acids		
Gallic acid	9.43 ± 0.47 a	10.20 ± 0.51 a
Protocatechuic acid	11.22 ± 0.56 a	4.38 ± 0.22 b
Chlorogenic acid	32.31 ± 1.62 a	7.34 ± 0.37 b
*p*-hydroxybenzoic acid	11.86 ± 0.59 a	5.85 ± 0.29 b
Vanillic acid	3.55 ± 0.18 a	2.03 ± 0.10 b
*p*-coumaric acid	nd ^2^	1.23 ± 0.06 a
Ferulic acid	nd	nd
Veratric acid	2.53 ± 0.13 a	nd
Benzoic acid	62.16 ± 3.11 a	27.97 ± 1.40 b
*t*-cinnamic acid	0.30 ± 0.02 a	0.23 ± 0.01 ab
Total	133.36	59.23
Flavonols		
Epigallocatechin	90.52 ± 4.53 ab	115.19 ± 5.76 a
Catechin	81.22 ± 4.06 a	21.30 ± 1.07 b
Epicatechin	9.59 ± 0.48 a	5.38 ± 0.27 b
Epigallocatechin gallate	21.83 ± 1.09 a	21.44 ± 1.07 a
Vanillin	nd	nd
Rutin	nd	nd
Catechin gallate	nd	nd
Quercetin	177.79 ± 8.89 a	112.94 ± 5.65 b
Naringin	1.87 ± 0.09 a	0.62 ± 0.03 b
Naringenin	5.37 ± 0.27 a	1.29 ± 0.06 b
Formonoetin	nd	nd
Total	388.19	278.16

^1^ All values are presented as the mean ± SD of pentaplicate determination. Different letters correspond to the significant differences related to the processing steps using Tukey’s multiple test (*p* < 0.05). ^2^ nd: not detected.

## Data Availability

All the data are available in the paper.
